# Diagnostic terminology used to describe atypia on breast core needle biopsy: correlation with excision and upgrade rates

**DOI:** 10.1186/s13000-019-0842-0

**Published:** 2019-06-29

**Authors:** Gary Tozbikian, Michael George, Debra L. Zynger

**Affiliations:** 1 Division of Breast Pathology, Department of Pathology, Wexner Medical Center at The Ohio State University, E414 Doan Hall, 410 W. 10th Ave, Columbus, OH 43210 USA; 2 Department of Medicine, University of Pittsburgh, Pittsburgh, PA USA; 3 Division of Genitourinary Pathology, Department of Pathology, The Ohio State University Wexner Medical Center, E401 Doan Hall, 410 W 10th Ave, Columbus, OH 43210 USA

**Keywords:** Breast, Atypical ductal hyperplasia, Atypia, Upgrade, Excision, Rate, Biopsy, Excision, Flat epithelial atypia

## Abstract

**Background:**

Subjective qualitative descriptors are sometimes used to describe atypical breast lesions diagnosed on core needle biopsy (CNB) which are limited in extent. In clinical practice, this terminology is used to imply a lower expected risk of upgrade on surgical excision (EXC). It is uncertain how subjective terminology impacts clinical management.

**Methods:**

We conducted a retrospective review of CNB with atypia and compared the EXC and upgrade rates of atypical ductal hyperplasia (ADH) and flat epithelial atypia (FEA) to lesions described as “focal” atypical ductal hyperplasia (FADH), to determine the impact of this diagnostic phrasing on surgical management and risk of malignancy.

**Results:**

FADH and ADH were excised at similar rates (82% vs. 78%). FADH lesions showed a similar upgrade rate (13%) compared to non-focal ADH (10%), and both showed a trend towards higher upgrade and EXC rates compared to FEA. ADH, FADH and FEA all had an upgrade risk that warranted EXC. In non-upgraded EXC, for each diagnostic category we observed similar rates of residual atypia in the EXC.

**Conclusions:**

Pathologists should avoid the use of qualitative descriptors when describing ADH on CNB because of the potential of this terminology to influence clinical decision making which is unwarranted.

## Background

Atypical ductal hyperplasia (ADH) is defined as an intraductal epithelial proliferation that has some but not all of the characteristics of low-grade ductal carcinoma in situ (DCIS), or which possesses all the features of DCIS but is limited in quantitative extent [1, 2]. Patients with ADH identified on needle core biopsy (CNB) have an increased risk for the subsequent development of both DCIS and invasive breast carcinoma [1–4]. ADH is considered a non-obligate precursor lesion that confers a 4–5 fold increased lifetime risk for invasive breast carcinoma [5, 6]. The diagnosis of ADH on CNB is generally an indication for surgical excision (EXC) [7–9], and is the recommendation per the National Comprehensive Cancer Network Guidelines based on the possibility that a more significant lesion could be missed due to sampling error [10]. In patients with ADH as the most significant finding on CNB that are treated by EXC, the reported upgrade rate (defined as the finding of DCIS or invasive carcinoma in the subsequent EXC) ranges from 11 to 87%, with studies using larger vacuum-assisted devices showing an upgrade rate of 15–30% [11–16]. Patients with ADH as the most significant finding after EXC may also be offered anti-endocrine chemopreventive therapy to reduce the risk of subsequent invasive and non-invasive breast cancer [17, 18]. However, anti-endocrine therapy is associated with significant risks such as increased vascular events and endometrial carcinoma [17]. Due to the morbidity associated with surgical intervention and anti-endocrine treatment, the accuracy of the diagnosis of ADH on CNB is critical.

There is a continued clinical interest in identifying low risk subsets of patients with ADH that can be conservatively managed by close observation instead of EXC. Published retrospective series have described a correlation between the quantitative extent of atypia of ADH and the risk of upgrade on subsequent EXC. [6, 7, 10] Lower upgrade rates have been reported in CNB with ADH involving less foci [11, 12, 19], fewer tissue cores [12], smaller sized of ducts [20], and smaller spans [15]. Prior investigations have correlated the upgrade rate of ADH to histomorphologic features [21] and use of confirmatory immunohistochemical stains [22]. Potential preanalytical predictors of upgrade include the method of radiographic screening [23], needle size and use of vacuum assistance [16]. To date, all studies have concluded that the upgrade rate of ADH, including those ADH lesions of relatively limited extent, remains too high to avoid EXC [7, 24, 25].

In clinical practice, qualitative descriptors are occasionally used by pathologists to indicate that an atypical breast lesion is limited in quantitative extent. The qualitative descriptor “focal” has been used in our institution to describe select atypical ductal hyperplasia lesions. While there is no standardized definition of focal atypical ductal hyperplasia (FADH), the term was employed by our pathologists to indicate an ADH lesion that was quantitatively limited in extent because either: 1) the atypical focus involved only a single duct space, 2) the quantitative amount of atypia was small relative to the total amount of tissue sampled in the CNB specimen, or 3) the lesion was present in one or two initial tissue levels, but was not present in the subsequent tissue levels. The term FADH was informally used to imply that there may not be residual atypia present in the anticipated EXC specimen, and the lesion may have a lower than expected risk of upgrade compared to a typical “non-focal” ADH lesion.

It is unknown how qualitative descriptors such as FADH impact clinical management. This is critical as the use of these terms may be subject to interobserver variability in usage. Importantly, the extent to which these descriptors influence clinical management has not been studied. In this analysis, we examined the impact of the use of the diagnosis FADH on rate of EXC and compared the upgrade rate of FADH to ADH and FEA. Additionally, correlations between excision and upgrade rates to relevant clinicopathologic variables including personal history of remote breast cancer, mammographic target, needle size and use of vacuum assistance, use of confirmatory immunostains (CK5 or CK5/6), presence of background lesions such intraductal papilloma or lobular neoplasia, pathologist and surgeon, were evaluated for each diagnostic category. We also correlated each diagnostic category with presence of residual atypia found in the EXC specimen, to see if FADH lesions were more likely to be entirely excised by the CNB compared to ADH and FEA.

## Methods

After approval by the institutional review board, we conducted a search of the pathology database for CNB performed at our institution from 2009 to 2015 with a diagnosis containing “atypical” or “atypia”. ADH cases in which the qualitative diagnostic word “focal” was used were categorized as a separate category. Three categories of atypia were compared: ADH, FADH, and FEA. If multiple atypical lesions were identified (e.g. ADH and FEA), the case was classified according to the most significant lesion (ADH > FADH > FEA). Cases were excluded if the diagnosis included invasive carcinoma or in situ carcinoma (either ductal or lobular carcinoma in situ). The use of CK5 or CK5/6 immunostaining as a marker of atypia was noted, excluding use as part of our institutional myoepithelial panel. Additional background pathologic lesions (e.g. intraductal papilloma (IDP), fibroepithelial lesion) or associated with atypical lobular hyperplasia (ALH) were documented. The pathologist that evaluated the biopsy was recorded.

The type of biopsy guidance modality (ultrasound vs. stereotactic vs. magnetic resonance imaging (MRI)-guided), mammographic target (mass vs. calcifications vs. enhancement), needle gauge, use of vacuum assistance, and presence of adequate radiology-pathology correlation were collected from the radiology report. A personal history of breast cancer and laterality (ipsilateral or contralateral to the biopsy showing atypia, as well as whether the lesion was synchronous or metachronous), and family history of breast cancer was obtained from the medical record. A synchronous personal history of breast cancer was defined as a history of breast cancer diagnosed within 6 months of the CNB, while metachronous was greater than 6 months. Patients with a history of synchronous ipsilateral breast cancer (invasive carcinoma or DCIS) were excluded if the definitive excision was not yet performed prior to the index CNB showing atypia (ADH, FADH, or FEA) because we would be unable to determine whether a finding of invasive cancer or DCIS on the excision represented a true upgrade.

Cases with ADH, FADH, and FEA as most significant diagnosis were correlated with findings on EXC. The pathology report from the corresponding EXC performed at our institution was reviewed and type of EXC (mastectomy vs. partial mastectomy), surgeon, and diagnosis was noted. Rate of upgrade was calculated for each CNB diagnostic category. An upgrade was defined as the presence of DCIS or invasive carcinoma identified in the EXC. The histologic slides from the CNB and EXC were re-reviewed in all upgraded cases. In EXC without upgrade, the presence of residual atypia (ADH or FEA) was recorded. Rate of excision was calculated for each CNB diagnosis category. If no EXC was performed, the reason was determined from the electronic medical record. Clinical followup was obtained for all by review of the pathology database.

Correlation between upgrade and excision rates versus diagnostic category was calculated via chi-square test. Significance of excision rates and rates of no residual atypia by diagnostic category were done by 3 × 2 chi-square test. Comparison of excision rates by surgeon and upgrade rates by pathologist were calculated by logistic regression with deviation from means coding. The significance of the association between upgrade rate to clinical and preclinical variables was evaluated by a logistic regression model using the diagnostic category and independent variable to predict upgrade. A *p*-value of ≤0.05 was considered statistically significant.

## Results

From 2009 to 2015, we identified 413 breast CNB specimens with ADH, FADH, or FEA as the most significant diagnosis. 79% (*n* = 327) had a subsequent EXC specimen at our institution [Table 1]. The mean age for the cohort was 54.8 years. Of those cases with EXC, the indications for CNB included mammographically detected microcalcifications (66%, *n* = 216), mass (28%, *n* = 92), MRI enhancement (4%, *n* = 14), and distortion/asymmetry (2%, *n* = 5). A total of 8 different pathologists reviewed either CNB or EXC specimens, with the majority of CNB (93%, *n* = 385) reviewed by 6 pathologists. A total of 6 different breast surgeons performed EXC.

**Table 1:** Excision, upgrade and residual atypia rates by diagnostic category

Diagnosis on CNB	Total n	Excised *n* (%)	*P*-value	Residual Atypia, No DCIS or IDC	Upgrade to DCIS	Upgrade to IDC	Overall Upgrade	*P*-value
ADH	205	169 (82)	–	93 (55)	13 (8)	4 (2)	17 (10)	–
FADH	129	101 (78)	–	47 (47)	8 (8)	5 (5)	13 (13)	–
FEA	79	57 (72)	–	34 (60)	2 (4)	1 (2)	3 (5)	–
Total	413	327 (79)	0.15	174 (54)	23 (7)	10 (3)	33 (10)	0.30

### Diagnostic category versus excision rate and upgrade rate

In this series, the overall excision rate for all categories of atypia was 79%. [Table 1]. There was no significant difference in the excision rate between ADH and FADH (82% vs. 78%, *p* = 0.4), or between the combined category of ADH/FADH and FEA (81% vs. 72%, *p* = 0.09).

**Fig. 1:**
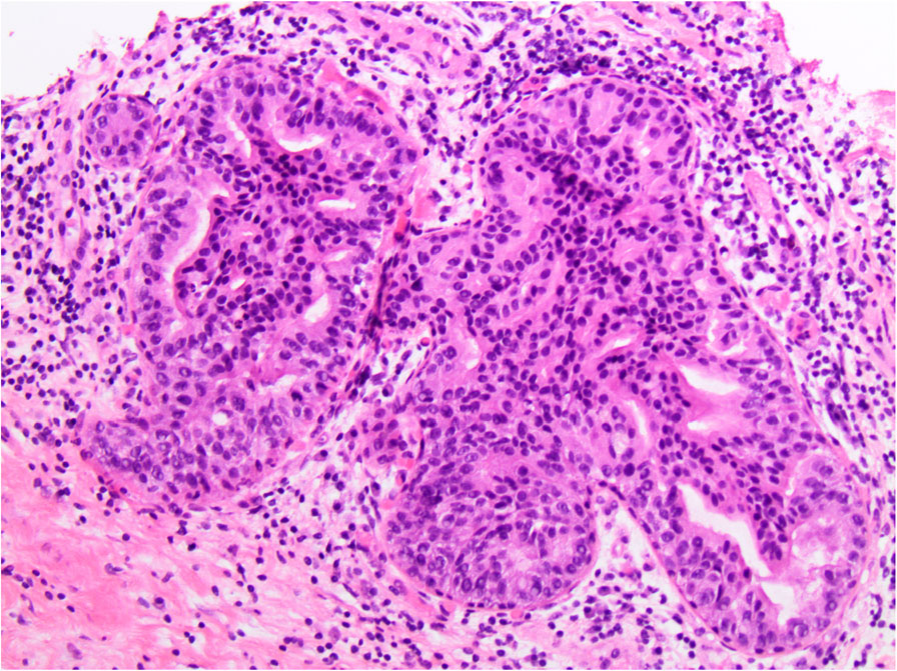
Patient #1 (H&E, 20x) a 59 year old woman with an ultrasound-guided core needle biopsy (CNB) with focal atypical ductal hyperplasia (FADH)

**Fig. 2:**
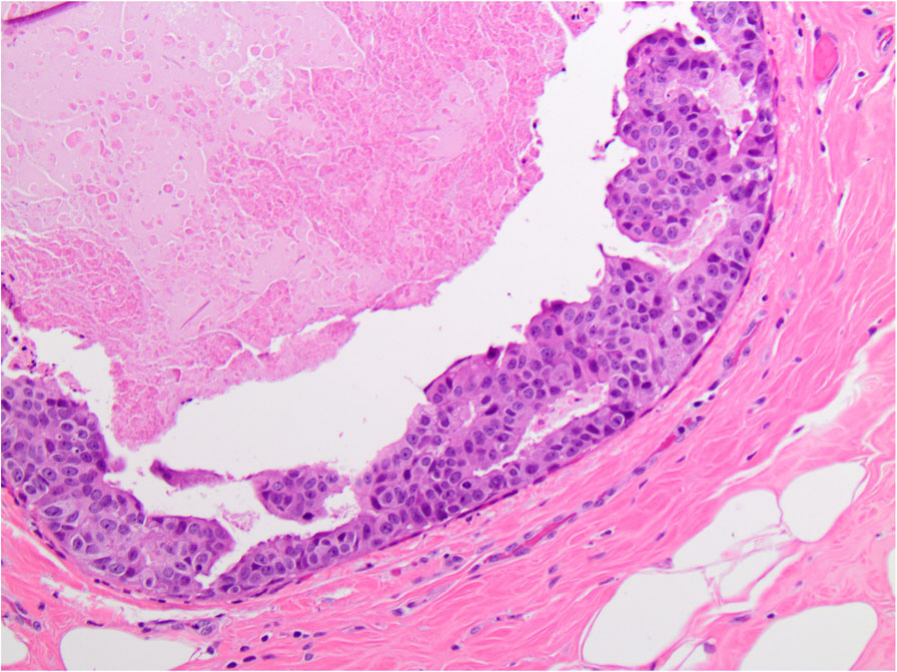
Patient #1 (H&E, 20x) the excision (EXC) showed ductal carcinoma in situ (DCIS)

**Fig. 3:**
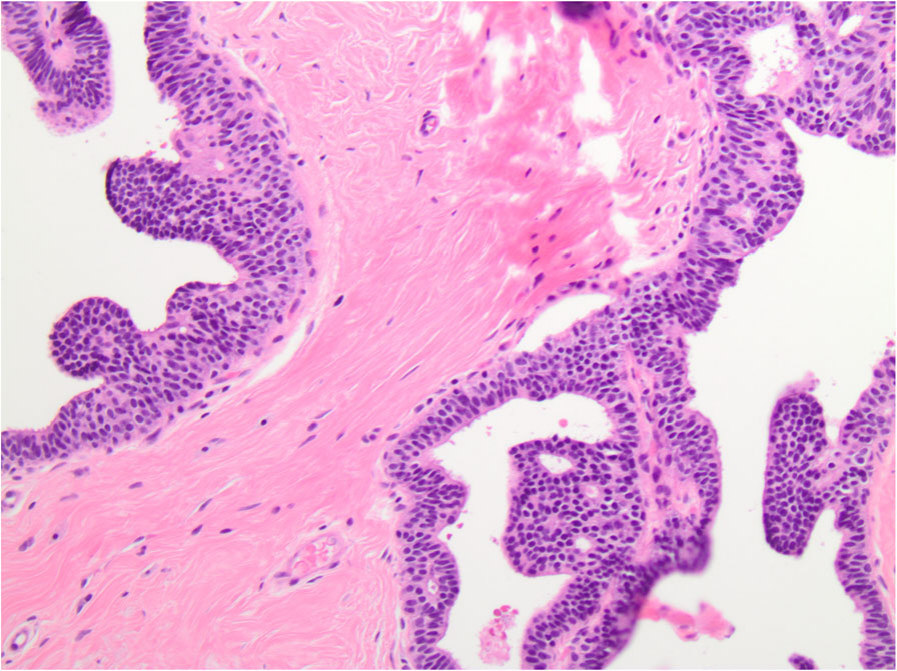
Patient #2 (H&E, 20x) a 52 year old woman with an ultrasound-guided CNB with atypical ductal hyperplasia (ADH)

**Fig. 4:**
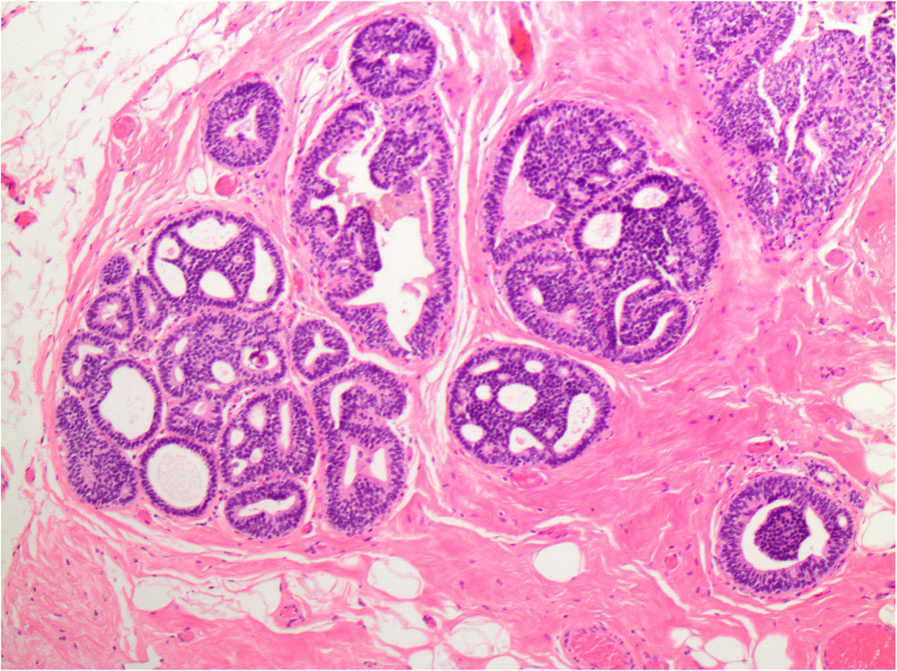
Patient #2 (H&E, 10x) EXC showed ductal carcinoma in situ (DCIS)

**Fig. 5:**
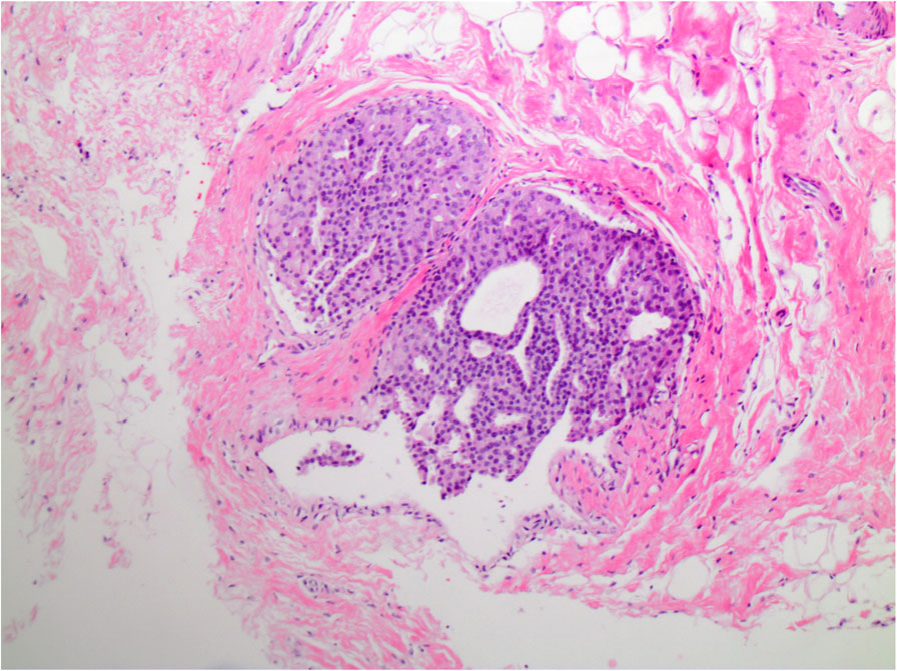
Patient #3 (H&E, 10x) a 52 year old woman with an ultrasound-guided CNB with focal atypical ductal hyperplasia (FADH)

**Fig. 6:**
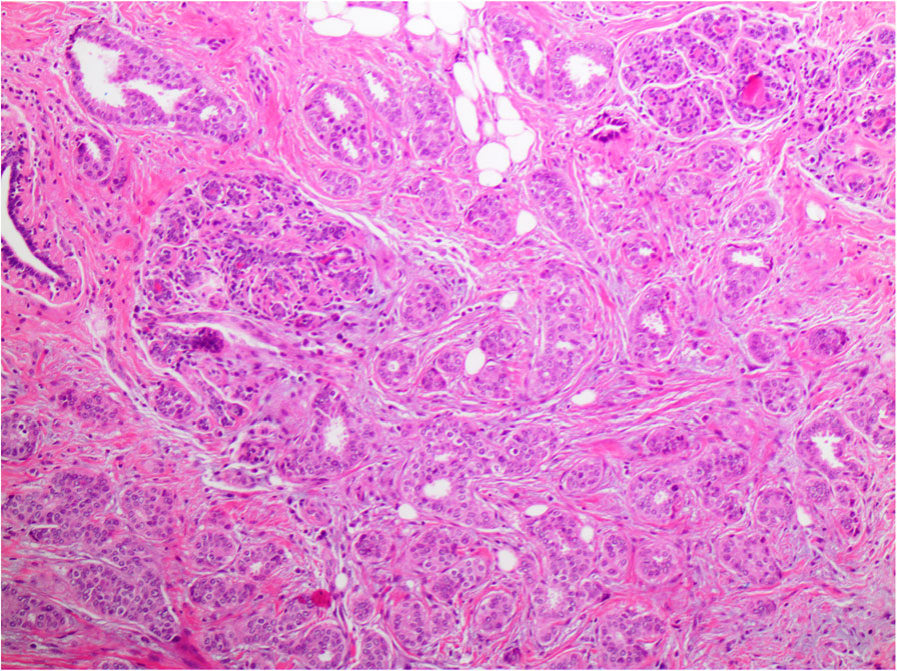
Patient #3 (H&E, 10x) EXC showed invasive ductal carcinoma

The overall frequency of upgrade to malignancy was 10% (33/327) [Table 1]. The majority of upgrades were to DCIS (7%, 23/327) [Figs. 1, 2, 3, and 4], with one third of the upgrades to invasive carcinoma (3%, 10/327) [Figs. 5 and 6]. There was no significant difference between the upgrade rates of FADH (13%, 13/101) versus ADH (10%, 17/169) (*p* = 0.55). ADH and FADH upgraded at approximately twice the rate of FEA (5%, 3/57), however the difference was not significant (*p* = 0.23) [Table 1]. Among EXC, a lack of residual atypia (absence of ADH or FEA, as well as no upgrade to invasive carcinoma or DCIS) indicating complete excision of the index lesion by the preceding CNB procedure was seen in 36% of cases, with no significant difference observed among the diagnostic categories (*p* = 0.46).

### Other variables versus excision rate and upgrade rate

Excision rate was significantly correlated with younger patient age. The mean age for patients who underwent EXC was 54.2 years (range: 30–79), was significantly younger than the mean of age of patients who did not receive an EXC (57.6 years, range: 25–89, *p* = 0.007). Patient age ≥ 50 status was not significantly different in patients who underwent EXC versus those who did not (65% vs. 72%, (*p* = 0.20). The incidence of family history of breast cancer was similar between patients who underwent EXC and those who did not (53% vs. 50%, *p* = 0.62). Rate of personal history of breast cancer was also similar between EXC vs. non-EXC cohorts (18% vs.12%, *p* = 0.19).. Among patients who underwent EXC, 5% (*n* = 15) had a personal history of breast atypia (ADH, ALH, or FEA), compared to 7% (*n* = 6) in patients who did not undergo EXC (*p* = 0.41). Radiology-pathology correlation was comparable for both patients who had EXC and those which did not (98% vs. 95%, *p* = 0.28).

Excision rates for each category of atypia were compared between surgeons. The average excision rate for all categories of atypia among all 6 surgeons was 79%, with a range of 71–94% We found significant individual variability in excision rates among the surgeons (*p* = 0.0002), with one surgeon showing significantly higher rate of EXC (*p* = 0.001), and another surgeon showing comparatively lower EXC rate compared to group (*p* = 0.001). Of 86 cases which were not excised, the reasons provided include: patient opted for close follow-up (*n* = 41), patient lost to follow-up (*n* = 21), physician recommendation of close clinical follow-up (*n* = 12), patient went to outside hospital for 2nd opinion (*n* = 6), non-specified (*n* = 4), and medical co-morbidities precluding surgery (*n* = 2). Only one reason for non-excision (patient opted for close followup) showed a correlation with surgeon provider, with 32/41cases from one surgeon (*p* = < 0.0001).

Upgrade rates were correlated with the presence or absence of clinical variables for all categories of atypia. Older patient age (≥50 years vs. < 50 years) was significantly associated with a higher upgrade rate (15% vs. 3%, *p* = < 0.001). Positive family history was also found to significantly increase the upgrade rate for all atypia (14% vs. 5%, *p* = 0.01). However, neither a personal history of breast cancer nor a history of prior diagnosis of atypia significantly correlated with upgrade rate. Neither radiologic target nor needle size (8–11 gauge vs. > 11 gauge) correlated with upgrade rate. Radiology-pathology correlation was achieved in the majority of cases (97%), and lack of radiology-pathology correlation was not significantly correlated with upgrade rate.

The use of CK5 or CK5/6 immunohistochemistry was utilized in 55% of cases and correlated with a significantly higher upgrade rate (24% vs. 9%, *p* = 0.01). Many of the CNB in this series were associated with background pathologic lesions (ALH in 8% and IDP in 8%), however the presence of these lesions was not significantly correlated with upgrade. The case pathologist that diagnosed the CNB did not impact the upgrade rate (*p* = 0.7).

Clinical followup was obtained on all patients. Synchronous contralateral carcinoma (invasive carcinoma or DCIS) was observed in 3% (*n* = 12) of patients. Metachronous carcinoma (invasive carcinoma, DCIS, or LCIS) was observed in 3% (*n* = 14) of patients. All metachronous carcinomas occurred between 1 to 4 years after an index diagnosis of either ADH (*n* = 13) or FADH (*n* = 1). Contralateral metachronous carcinoma (3 cases of invasive carcinoma, 3 cases of DCIS, and 2 cases of LCIS) was slightly more frequent than ipsilateral metachronous carcinoma (3 cases of invasive carcinoma and 3 cases of DCIS). The majority of metachronous carcinoma (*n* = 11) occurred in patients who underwent EXC for their index atypical lesion, whereas 3 cases of metachronous carcinoma (1 contralateral, 2 ipsilateral) occurred in patients who did not undergo EXC of their index atypical lesion.

## Discussion

Patients who have ADH diagnosed on CNB are recommended to undergo EXC due to the high risk of upgrade to malignancy. This approach to the management of patients with ADH is supported by the American College of Radiology Breast Imaging Reporting and Data Systems (BIRAD) Atlas which recommends followup for breast imaging findings with a risk of malignancy of less than 2% (BIRADS 3) [26]. Since all studies have shown that the risk of upgrade of ADH lesions is greater than this 2% threshold, EXC is generally warranted [11–16]. However, the majority of patients who undergo EXC for ADH do not upgrade. Due to the morbidity of EXC many investigators have examined various clinical, radiologic, and pathologic variables to identify those with discriminatory power to risk stratify patients and identify a low risk subset who can safely avoid EXC. Multiple studies have retrospectively analyzed CNB specimens with a diagnosis of ADH and correlated the quantitative extent of atypia present with the risk of upgrade on subsequent EXC and have found comparatively higher upgrade rates in more extensive ADH lesions [11, 12, 19, 24, 25]. Several measures of the quantitative extent of atypia are included in a recently published nomogram that risk-stratifies patients with ADH on CNB as a clinical tool to assist treating surgeons considering EXC. [15] However in routine clinical practice, most pathology reports of atypical ductal hyperplasia do not provide specific details regarding the extent of atypia (e.g. number of involved tissue cores, number of involved ducts, or linear extent of atypia) thus limiting the practical utility of such nomograms outside of the scope of a research setting. In routine practice, pathologists may provide more generalized descriptions of the quantitative extent of atypia by using summative diagnostic descriptors such as “focal” to describe ADH lesions seen on CNB. To date, no studies have correlated diagnostic descriptors used in clinical practice with risk of upgrade of atypical breast lesions, nor examined the potential impact of such descriptive terminology on clinician decision-making, and specifically the surgical EXC for these patients.

We did not identify that FADH was excised at a lower rate than ADH in our institution. Therefore, our cohort is appropriate to compare upgrade risks between these groups. In our series, we observed no significant difference in upgrade risk for ADH lesions which were qualified as being focal (13%) vs. non-focal (9%). Our results are similar to several other studies which demonstrate a risk of upgrade for all ADH lesions, regardless if the lesion was noted to be limited in quantitative extent (< 3 foci) [11, 12, 24, 25, 27] or microscopic span [28, 29], that is too high to avoid surgical excision. Our study also underscores the findings of the recent Nurses Health Studies that demonstrated that patients with ADH of limited extent (defined as < 3 foci) showed similar lifetime risk of developing subsequent breast cancer compared to women with more extensive ADH lesions [30]. This inability to identify a quantitative threshold that reduces risk of upgrade below 2%, as well as lack of demonstrable dose-response relationship between extent of atypia and risk of breast cancer, indicate that the histopathologic extent of atypia should not be used to inform clinical management decisions for patient diagnosed with ADH. Descriptive terminology such as “focal” to qualify ADH lesions found on CNB should be avoided in clinical practice, as it could be incorrectly interpreted as a lower risk lesion.

Lack of interobserver reproducibility between pathologists diagnosing ADH has been reported, with upgrade rates and diagnostic concordance shown to correlate with pathologists’ level of expertise, case volume, and practice setting [16, 31]. The study pathologists participating in this investigation were from a fully subspecialized, high-volume academic medical center, and upgrade rates were similar among all pathologists, indicating minimal clinically impactful analytical variability. We also noted a significant correlation of upgrade rate with older age and family history of breast cancer in our study population. The increasing risk of atypia upgrade with patient age and family history of breast cancer that we noted is consistent with well-established published data [29, 32, 33]. Other findings in our analysis are interesting to note. The excision rate was higher among younger patients in our series, despite a significantly lower upgrade risk. While multiple reasons could potentially explain this trend, (e.g. patient co-morbidities and other clinical factors limiting surgical options) the majority reason for non- EXC was due to the patients’ desire to pursue close clinical followup. We also noted significant variation in the EXC rates among the 6 surgeons in our series. Our data does suggests a large role of individual differences in practice patterns and patient counseling by the surgeon, as some surgeons had very high EXC rates, while the majority of patients electing for close clincial followup were concentrated from a single surgeon.

## Conclusions

Pathologists should limit the use of qualitative descriptors when describing atypical lesions on core biopsy. The extent of atypia does not influence upgrade rate in a meaningful way, while this terminology did not appear to impact surgical decision-making in our study, the potential for such terminology to cause confusion to surgeons and improperly influence decision-making argues against the use of this diagnostic phrasing in routine pathology practice.

## Data Availability

Data and materials of this work are available from the corresponding author on reasonable request.

## Abbreviations

ADH: Atypical ductal hyperplasia
ALH: Atypical lobular hyperplasia
BIRAD: Breast Imaging Reporting and Data Systems
CNB: Core needle biopsy
DCIS: Ductal carcinoma in situ
EXC: Excision
FEA: Flat epithelial atypia
IDP: Intraductal papilloma
MRI: Magnetic resonance imaging
